# Characterization of an Emergent Chicken H3N8 Influenza Virus in Southern China: a Potential Threat to Public Health

**DOI:** 10.1128/jvi.00434-23

**Published:** 2023-06-08

**Authors:** Peiwen Chen, Ziying Jin, Liuxia Peng, Zuoyi Zheng, Yiu-Man Cheung, Jing Guan, Liming Chen, Yiteng Huang, Xiaohui Fan, Zengfeng Zhang, Dongmei Shi, Jin Xie, Rirong Chen, Boheng Xiao, Chun Hung Yip, David K. Smith, Wenshan Hong, Yongmei Liu, Lifeng Li, Jia Wang, Edward C. Holmes, Tommy Tsan-Yuk Lam, Huachen Zhu, Yi Guan

**Affiliations:** a Guangdong-Hong Kong Joint Laboratory of Emerging Infectious Diseases, Joint Institute of Virology (STU/HKU), Shantou University, Shantou, Guangdong, China; b State Key Laboratory of Emerging Infectious Diseases (SKLEID), School of Public Health, Li Ka Shing Faculty of Medicine, The University of Hong Kong, Hong Kong SAR, China; c Department of Microbiology, Guangxi Medical University, Nanning, Guangxi, China; d The First Affiliated Hospital, Shantou University Medical College, Shantou, Guangdong, China; e Advanced Pathogen Research Institute, Shenzhen, Guangdong, China; f Laboratory of Data Discovery for Health Limited, Hong Kong SAR, China; g Sydney Institute for Infectious Diseases, School of Medical Sciences, University of Sydney, Sydney, New South Wales, Australia; Emory University School of Medicine

**Keywords:** ferret, H3N8, influenza, transmissibility, pathogenesis, chicken, infectivity, zoonosis

## Abstract

Although influenza A viruses of several subtypes have occasionally infected humans, to date only those of the H1, H2, and H3 subtypes have led to pandemics and become established in humans. The detection of two human infections by avian H3N8 viruses in April and May of 2022 raised pandemic concerns. Recent studies have shown the H3N8 viruses were introduced into humans from poultry, although their genesis, prevalence, and transmissibility in mammals have not been fully elucidated. Findings generated from our systematic influenza surveillance showed that this H3N8 influenza virus was first detected in chickens in July 2021 and then disseminated and became established in chickens over wider regions of China. Phylogenetic analyses revealed that the H3 HA and N8 NA were derived from avian viruses prevalent in domestic ducks in the Guangxi-Guangdong region, while all internal genes were from enzootic poultry H9N2 viruses. The novel H3N8 viruses form independent lineages in the glycoprotein gene trees, but their internal genes are mixed with those of H9N2 viruses, indicating continuous gene exchange among these viruses. Experimental infection of ferrets with three chicken H3N8 viruses showed transmission through direct contact and inefficient transmission by airborne exposure. Examination of contemporary human sera detected only very limited antibody cross-reaction to these viruses. The continuing evolution of these viruses in poultry could pose an ongoing pandemic threat.

**IMPORTANCE** A novel H3N8 virus with demonstrated zoonotic potential has emerged and disseminated in chickens in China. It was generated by reassortment between avian H3 and N8 virus(es) and long-term enzootic H9N2 viruses present in southern China. This H3N8 virus has maintained independent H3 and N8 gene lineages but continues to exchange internal genes with other H9N2 viruses to form novel variants. Our experimental studies showed that these H3N8 viruses were transmissible in ferrets, and serological data suggest that the human population lacks effective immunological protection against it. With its wide geographical distribution and continuing evolution in chickens, other spillovers to humans can be expected and might lead to more efficient transmission in humans.

## INTRODUCTION

An important lesson from the 2009 A(H1N1) influenza pandemic was that a zoonotic virus could still trigger a pandemic even if it was of the same subtype as current seasonal viruses (1, 2). In the last three decades several virus lineages, including H5Nx, H9N2, and H7N9, have become established in poultry in China (3–6). All seem to have pandemic potential, as they have been repeatedly introduced into humans, providing new evidence to support the hypothesis that China is the “epicenter” of new pandemic strains given its complex influenza ecosystem (7).

Over the last decade, several new zoonotic influenza viruses (e.g., H7N9, H10N8/N3) had similar emergence pathways with their internal gene cassettes being derived from enzootic H9N2 viruses (5, 8–11). Long-term enzootic viruses, such as those with the Goose/Guangdong/96-lineage H5, have also acquired internal genes from the H9N2 lineage and became predominant in the field, increasing opportunities to transmit to humans (12). If a novel virus bearing H9N2 internal gene constellations becomes prevalent in chickens in the field, spillovers to humans, as observed in the H7N9/2013, H10N8/2014, and H10N3/2021 viruses, appear likely. As part of pandemic influenza preparedness, novel emerging influenza viruses of this type should be considered pandemic threats and rapidly assessed.

The most recent example of an H9N2 internal gene-containing virus is the emergence of a novel H3N8 influenza virus, which was only identified after a human infection was detected (13 to 17). Based on accumulated experience over the last two decades in southeastern Asia, human infections with novel influenza viruses were only able to occur when there were large outbreaks of the viruses in animal populations (10, 12). While previous studies have confirmed the human H3N8 viruses were of avian origin, the details of their genesis and prevalence in the field need further exploration (14, 16). More importantly, the transmissibility and infectivity of the avian H3N8 in ferrets, the model for human transmissibility, have not been evaluated.

H3 influenza viruses have the broadest host species distribution among influenza viruses and have circulated in humans for almost 60 years (18–20). H3 is one of only three influenza virus subtypes known to cause pandemics and persist in humans (21, 22). The possibility of a new pandemic emerging from an animal H3 virus needs to be guarded against.

Here, we report results from our systematic influenza surveillance in southern China and demonstrate that the novel H3N8 virus has been prevalent in chickens for at least a year. Infection and transmission studies in ferrets showed that the H3N8 virus transmitted efficiently between ferrets through direct contact but inefficiently through airborne exposure. Serological studies indicated that the human population would have limited immunity if infected by these viruses. With the persistence and ongoing evolution of these viruses in chickens, the combination of their mammalian transmissibility and the limited human immunity to them raises the potential for sustained human-to-human transmission to develop. Thus, our findings suggest that influenza viruses with pandemic potential will continue to emerge from southern China.

## RESULTS

### Isolation of H3 influenza viruses from chickens.

**FIG 1:**
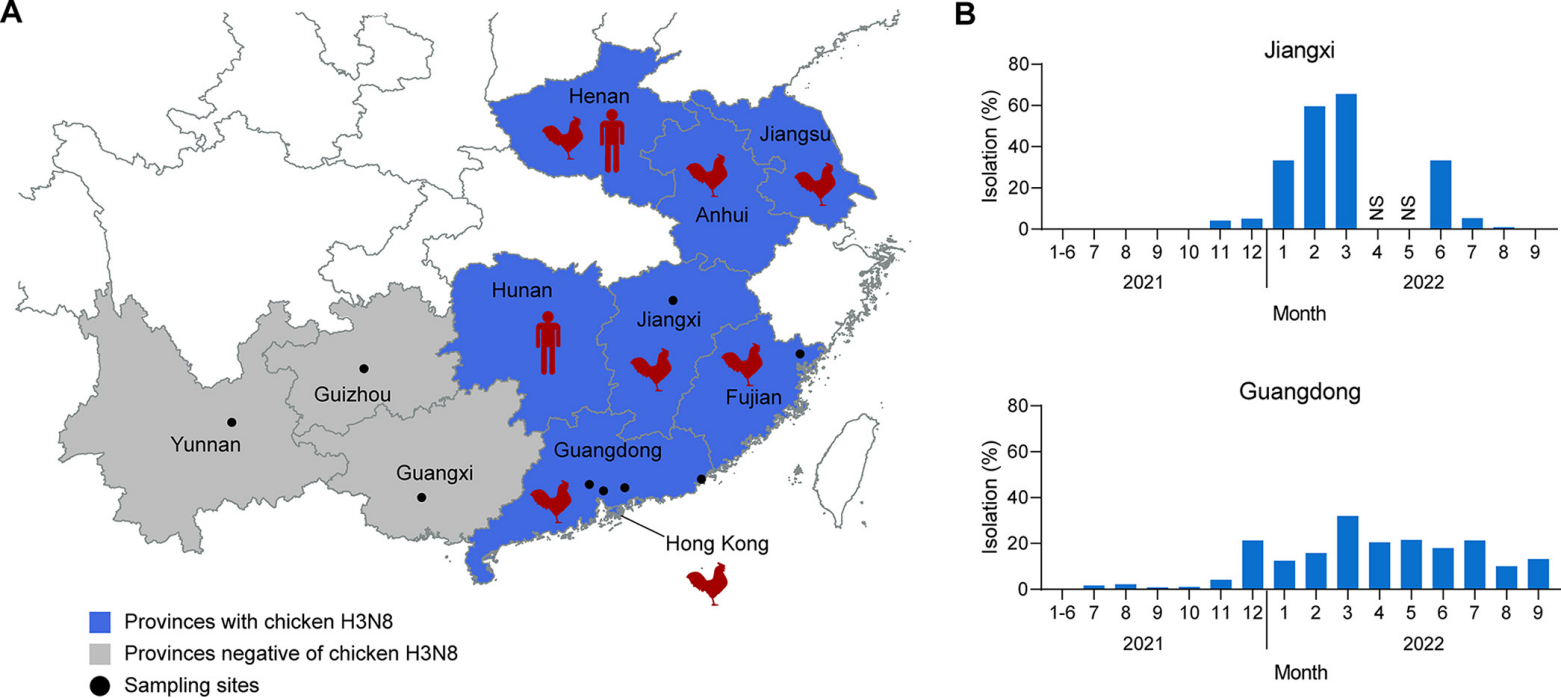
Distribution of H3N8 influenza virus and isolation of H3 subtype viruses in Guangdong and Jiangxi. (A) The location of the cities in China that reported the two H3N8 human infection cases are highlighted by a red human figure (13, 17). Blue shading and gray shading indicate the provinces that were positive and negative, respectively, for H3N8 viruses (data from this work and the previous studies of Yang et al. [15] and Sit et al. [16]). Black dots denote cities where our sampling occurred. The four cities surveyed in Guangdong province were Guangzhou, Dongguan, Huizhou, and Shantou, from left to right. Shape file for the China boundary was downloaded from the Stanford Digital Repository. (B) Monthly isolation rates of H3 influenza viruses in Guangdong and Jiangxi. NS, not sampled.

A total of 44,079 oropharyngeal or cloacal swabs of chickens were collected at live poultry markets in six Chinese provinces (Jiangxi, Guangdong, Guangxi, Fujian, Guizhou, and Yunnan) from January 2021 to September 2022 (Fig. 1A and Table 1). A total of 19,279 hemagglutinin-positive agents were isolated (43.7% of samples), of which 2,959 (6.7% of samples) were H3 and 15,331 (34.8% of samples) were H9 influenza A viruses (Table 1). The remaining hemagglutinin-positive isolates were other subtypes of influenza A viruses or avian paramyxoviruses.

**TABLE 1:** Isolation of H3 influenza virus in chickens from 2021 to 2022

Province^*a*^	Year	Month	Sample	No. of isolates			
				Flu A	H3^*b*^ (%) | seq.^*c*^	H9	Hx^*d*^
JX	2021	1–9	3,092	1,875	1 (0.0) | 1	1,867	7
		10	396	252	2 (0.5) | 2	247	3
		11	532	310	22 (4.1) | 6	239	49
		12	320	269	16 (5.0) | 12	249	4
	2022	1	388	303	129 (33.3) | 25	163	11
		2	320	259	191 (59.7) | 40	57	11
		3	160	123	105 (65.6) | 21	13	5
		4–5	/^*e*^	/	/	/	/
		6	306	251	102 (33.3)	143	6
		7	400	343	21 (5.3)	314	8
		8	322	252	3 (0.9)	249	0
		9	321	249	0 (0.0)	249	0
Subtotal			6,557	4,486	592 (9.0) | 107^*f*^	3,790	104
GD	2021	1–6	7,491	2,453	0 (0.0)	2,425	28
		7^*g*^	1,373	587	23 (1.7) | 18	557	7
		8	1,751	943	39 (2.2) | 39	890	14
		9	918	596	8 (0.9) | 8	583	5
		10	1,241	734	12 (1.0) | 12	699	23
		11	1,219	740	51 (4.2) | 29	670	19
		12	1,217	796	259 (21.3) | 97	521	16
	2022	1	956	378	119 (12.5) | 54	243	16
		2	1,073	503	169 (15.8) | 40	316	18
		3	1,277	748	407 (31.9) | 60	320	21
		4	1,212	845	249 (20.5) | 29	573	23
		5	1,192	785	258 (21.6)	507	20
		6	1,285	939	231 (18.0)	696	12
		7	1,153	564	186 (16.1)	365	13
		8	1,306	837	131 (10.0)	692	9
		9	1,189	859	157 (13.2)	673	29
Subtotal			25,853	13,307	2,299 (8.9) | 386^*h*^	10,730	273
GX	2021−2022		2,791	144	2 (0.1) | 2^*i*^	141	1
FJ	2021−2022		2,503	615	1 (0.0) | 1^*j*^	601	13
GZ	2021−2022		3,310	660	65 (2.0) | 38^*k*^	36	559
YN	2021−2022		3,065	67	0 (0.0)	33	34
Total			44,079	19,279	2,959 (6.7) | 534	15,331	984

a Provinces: JX, Jiangxi; GD, Guangdong; GX, Guangxi; FJ, Fujian; GZ, Guizhou; YN, Yunnan.

b The isolation rate of H3 is shown within parentheses.

c The number of H3 isolates selected for sequencing (seq.).

d Hx: H4, H5, H6, H8, H10, H11, and mixed infection.

e /, Not sampled.

f 105 H3N8 and 2 H3N2.

g H3N8 virus was first detected on July 2021 in GD.

h 382 H3N8, 1 H3N2, 1 H3H9N2, and 2 H6.

i–k 41 H3N2.

H3 influenza viruses were present in seemingly healthy chickens at live-poultry markets (LPMs) from July 2021 to September 2022 (the end of this surveillance) in Guangdong and from October 2021 onwards in Jiangxi (Fig. 1B and Table 1). In some months, H3 viruses were isolated at higher frequencies than enzootic H9N2 viruses and a total of 2,891 H3 viruses were identified from these provinces (Fig. 1B and Table 1). In the remaining four sampled provinces, only 68 H3 influenza viruses were isolated; 41 were selected for sequencing and were of the H3N2 subtype (Table 1). Selected H3 isolates (*n* = 493) from Guangdong and Jiangxi were also confirmed to be H3N8 viruses by genome sequencing. A total of 489 H3N8 isolates were obtained and sequenced from Guangdong and Jiangxi. Of these, 215 were identified as mixed infections involving multiple subtypes of influenza viruses, while the remainder, 274, were H3N8 viruses.

### Phylogenetic analysis of chicken H3N8 viruses.

**FIG 2:**
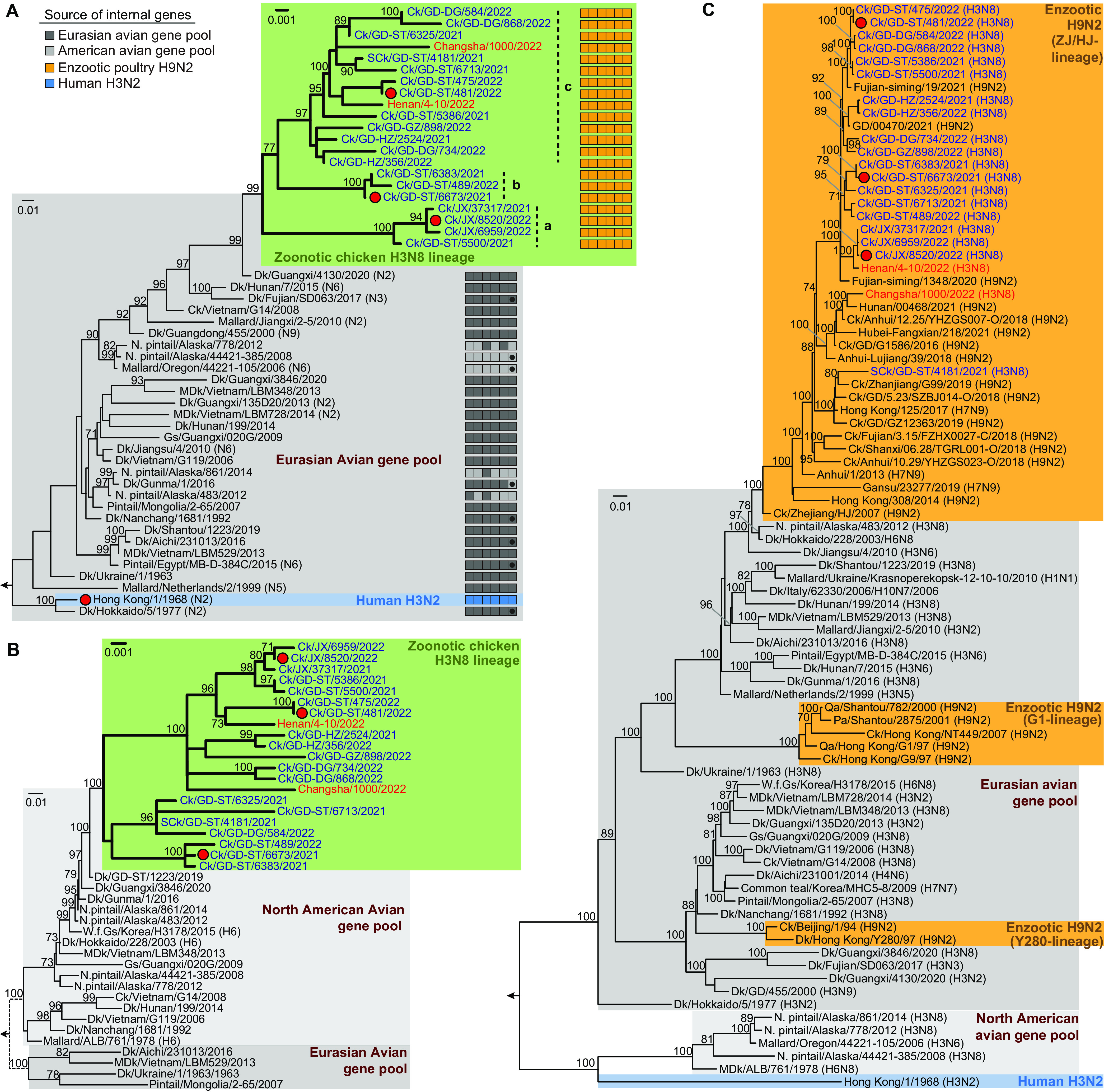
Maximum likelihood phylogenies of selected H3 hemagglutinin (*n* = 60) (A), N8 neuraminidase (*n* = 50) (B), and PB2 (*n* = 92) (C) genes. Full trees are given in Fig. S1 and S2. The recent human H3N8 viruses are labeled in red, and the chicken viruses isolated in this study are in blue. Viruses used in the animal experiments are indicated by red circles. Hong Kong/1/1968, the prototype human H3N2 pandemic virus, has a blue background. Lineage sources of the six internal gene segments are indicated by the colors of the boxes (left to right: PB2, PB1, PA, NP, M, and NS) to the right of the tree, and dots in the NS box indicate allele B (A). Viruses of the chicken H3N8 lineage are highlighted with a green background and their subtree is magnified by ×10 relative to the rest of the tree (A and B). Gray backgrounds indicate viruses from the avian gene pool, with Eurasian viruses in darker gray and North American viruses in light gray. Orange boxes in the PB2 phylogeny (C) highlight viruses from enzootic H9N2 lineages in China. Numbers at nodes indicate topological support as assessed by 1,000 bootstrap replicates. The length of the scale bar corresponds to 0.01 nucleotide substitution per site. (Host species: Ck, chicken; SCk, silkie chicken; Dk, duck; Qa, quail; Pa, partridge; Gs, goose; W.f. Gs, white-fronted goose; N. pintail, Northern pintail; MDk, muscovy duck). Geography: Chinese province: JX, Jiangx; GD, Guangdong; cities in Guangdong province: ST, Shantou; DG, Dongguan; GZ, Guangzhou; HZ, Huizhou.

Full genome sequences of the 274 H3N8 single virus isolates were combined with Eurasian gene pool viruses for phylogenetic analysis. The H3 HA and N8 NA genes were derived from viruses circulating in domestic ducks in southern China (Fig. 2A and B), and all six internal genes were obtained from the enzootic H9N2 (Ck/Zhejiang/HJ/2007-like) virus lineage (Fig. 2C and Fig. S1 and S2 in the supplemental material) (5, 23). In the H3 HA phylogeny, the novel chicken and the two human H3N8 viruses formed a monophyletic group, most closely related to Dk/Guangxi/4130/2020 (H3N2), and formed three clades denoted *a*, *b,* and *c* (Fig. 2A and Fig. S1). Clade *a* was mainly comprised of viruses circulating in chickens from Jiangxi, while the remaining two clades mostly contained viruses from Guangdong. The two recent human viruses from Henan and Hunan provinces were in clade *c*. In the N8 NA phylogeny, all novel chicken H3N8 viruses were also monophyletic, forming two clades, one of which contained viruses from Guangdong and Jiangxi and the two human isolates, while the other consisted of viruses exclusively from Guangdong (Fig. 2B and Fig. S1). The N8 gene of the chicken H3N8 viruses was most closely related to Dk/Shantou/1223/2019 (H3N8). For the six internal genes, all formed clades with the recent enzootic H9N2 viruses circulating in different regions, suggesting frequent reassortment between the H3N8 and H9N2 viruses (Fig. 2C and Fig. S1 and S2). Taken together, phylogenetic data suggest that the chicken H3N8 virus was likely generated after duck H3N8 and/or H3N2 viruses were introduced to chickens and then reassorted with enzootic H9N2 viruses.

### Molecular characteristics of the novel H3N8 viruses.

All the chicken H3N8 isolates have 193N, 222W, 226Q, 227S, and 228G (H3 numbering) in the receptor binding domain (RBD) of the HA, as does one of the two human H3N8 isolates (Changsha/1000/2022). These markers are associated with the ability to bind either α-2,3-linked or α-2,6-linked sialic acids in H3 subtype viruses (15). The other human isolate, Henan/4-10/2022, had partially switched to S at the HA-228 position. No deletion was found in the NA stalk region of the chicken H3N8 isolates and the two human isolates. Several substitutions related to mammalian adaptation or enhanced activity of the viral polymerase, such as 292V, 588V, and 702R in PB2, 368V and 473V in PB1, 356R and 409N in PA, and 172K and 205S in NS1, were found in most of the chicken H3N8 viruses. Notably, the PB2 627K (found in Henan/4-10/2022 but not Changsha/1000/2022) and 701N mammalian markers were absent (Table S1). The recognized substitutions associated with decreased pH for activation of the hemagglutinin were not identified in the chicken H3N8 isolates of our study and also the two human isolates (Table S1).

### Infectivity, transmissibility, and pathogenicity of chicken H3N8 viruses in ferrets.

To evaluate the possible risk of this novel H3N8 virus to humans, we examined its infectivity, transmissibility, and pathogenicity in ferrets. Three H3N8 chicken viruses A/Ck/Guangdong-Shantou/481/2022 (Ck/481), A/Ck/Jiangxi/8520/2022 (Ck/8520), and A/Ck/Guangdong-Shantou/6673/2021 (Ck/6673) were selected from different regions and clades (Fig. 2A) for testing, along with a prototype H3N2 pandemic virus A/Hong Kong/1/1968 (HK1/68).

Influenza seronegative ferrets (*n* = 24, 6 per virus) were inoculated intranasally with a 10^6^ tissue culture infectious dose (TCID_50_) of each virus. Most ferrets inoculated with the H3N8 or HK1/68 viruses had a brief fever at 1 to 2 days postinoculation (dpi), demonstrated reduced activity and sneezing, had nasal discharges throughout the experiment (Table 2 and Fig. S3A, C, D, and E), and had minor loss of body weight (Table 2 and Fig. S3B). Viable virus could be detected from nasal washes of ferrets inoculated with the H3N8 viruses as early as 1 dpi and up to 6 dpi with titers ranging from 10^3^ to 10^7^ TCID_50_/mL (Fig. S3F). For ferrets infected with the H3N8 viruses, virus shedding was similar to those infected with HK1/68 for the first 3 days but was lower on 4 to 6 dpi (Fig. S3F). For the final 3 days, the average virus titers of the H3N8 or H3N2 inoculated ferrets were 10^4.5^ TCID_50_/mL for Ck/481, 10^3.7^ TCID_50_/mL for Ck/8520, 10^4.7^ TCID_50_/mL for Ck/6673, and 10^5.7^ TCID_50_/mL for HK1/68. Overall, the signs of infection with, and virus replication profiles of, the chicken H3N8 and HK1/68 viruses were similar in the ferret model.

**TABLE 2:** Clinical symptoms, virus detection, and seroconversion of ferrets inoculated with H3 influenza viruses

Virus	Clinical symptoms				Mean peak titer in NW^*e*^	Seroconverted^*f*^
	Fever^*a*^	Res.sym.^*b*^	RII^*c*^	Weight loss (%)^*d*^		
Ck/481	4/6	6/6	1.54	4.25	6.71	6/6 (320–1,280)
Ck/8520	6/6	6/6	1.52	4.59	6.70	6/6 (160–1,280)
Ck/6673	3/6	6/6	1.14	1.45	6.23	6/6 (160–640)
HK1/68	4/6	6/6	1.19	5.15	6.80	6/6 (640–1,280)

a Number of ferrets that exhibited fever (body temperature ≥1.5°C above baseline).

b Number of ferrets that displayed respiratory symptoms (Res.sym.), including nasal discharge, sneezing, and cough.

c Relative inactivity index (RII), scored as 0 to 3, monitored over 14 days (see Materials and Methods).

d Mean maximum weight loss, as a percentage of initial body weight, of six ferrets.

e Mean peak viral titers in nasal wash (NW) are expressed as log_10_ TCID_50_/mL.

f Number of ferrets that seroconverted with their range of HI titers in parentheses.

**FIG 3:**
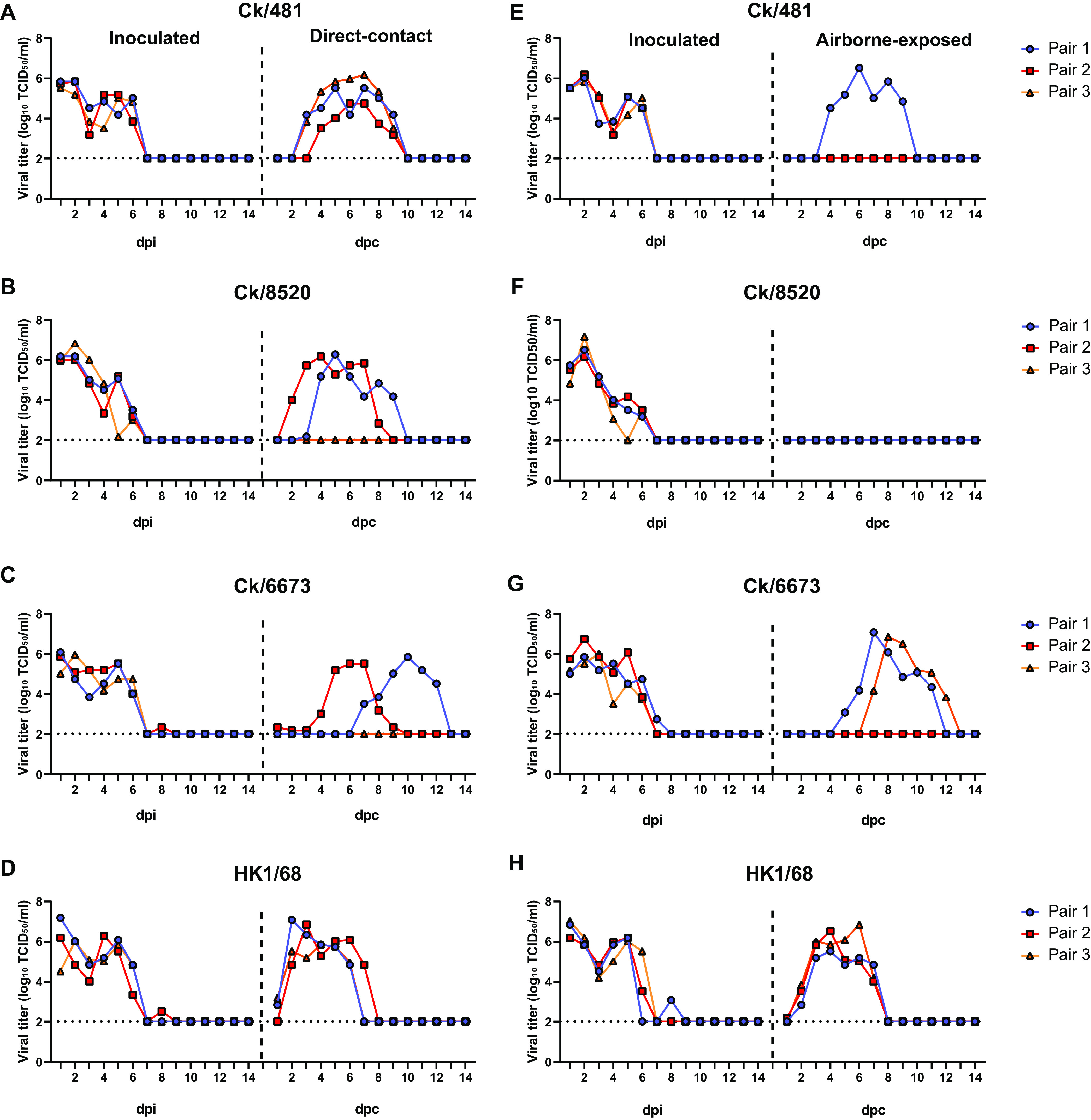
Transmissibility of H3N8 and H3N2 viruses in ferrets. Donor ferrets (*n* = 3) were inoculated with 10^6^ TCID_50_ of Ck/481 (A and E), Ck/8520 (B and F), Ck/6673 (C and G), and HK1/68 (D and H). Naïve ferrets were introduced at 1 dpi. The efficiency of direct-contact (A to D) and airborne (E to H) transmission was confirmed by detecting viruses in nasal washes. Nasal washes were collected daily (for 14 days) from each ferret and titrated in MDCK cells. In each subfigure, the left set of graphs is from inoculated ferrets and the right set is from naive ferrets. The detection limit is 2.02 log_10_ TCID_50_/mL; dpc, days postcontact.

For each virus, transmission from an infected donor animal to a direct-contact animal and from an infected donor animal to an airborne contact animal (each with three replicates) was evaluated (Fig. S4). Virus transmission was determined by titration of nasal washes and testing for seroconversion at 21 days postcontact (dpc). The HK1/68 virus was detected in nasal washes of all contact ferrets exposed by direct-contact and airborne routes (Table 3). Ferrets began shedding virus at 1 or 2 dpc, and this persisted in nasal washes for 6 days, with a peak virus titer >10^6^ TCID_50_/mL (Table 3 and Fig. 3D and H). All the contact ferrets exhibited respiratory symptoms and had seroconverted by 21 dpc, with hemagglutinin inhibition (HI) titers ≥1,280 (Table 3). Compared to HK1/68, fewer contact animals were infected by H3N8 viruses. In the direct-contact group, the transmission of the Ck/481, Ck/8520, and Ck/6673 viruses was observed in 3/3, 2/3, and 2/3 contact animals, respectively. The time to the transmission was generally delayed (ranging from 0 to 6 days later as determined by the first day of virus detection) and more variable than seen with HK1/68 (Table 3 and Fig. 3A to C). The direct-contact ferret exposed to Ck/8520 that did not shed detectable virus seroconverted at 21 dpc (Table 3). For the airborne exposure group, virus shedding in Ck/481, Ck/8520, and Ck/6673 groups were detected in 1/3, 0/3, and 2/3 contacts animals, respectively (Table 3 and Fig. 3E to G). In one ferret exposed to Ck/481, virus shedding was not detected, but seroconversion by 21 dpc was observed (Table 3).

**TABLE 3:** Transmission of H3N8 and H3N2 influenza viruses in ferrets

Virus	Direct-contact group					Airborne-exposed group				
	Res.sym.	No. of ferrets shed virus	Onset (dpc)^*a*^	Duration (days)	Seroconverted	Res.sym.	No. of ferrets shed virus	Onset (dpc)	Duration (days)	Seroconverted
Ck/481	3/3	3/3	3,3,4	7,7,6	3/3 (320, 640, 640)	1/3	1/3	4,/,/	6,/,/	2/3 (160, 160, <10)
Ck/8520	2/3	2/3	2,3,/	7,7,/	3/3 (320, 320, 640)	0/3	0/3	/,/,/	/,/,/	0/3 (<10, <10, <10)
Ck/6673	2/3	2/3	4,7,/	6,7,/	2/3 (640, 640, <10)	2/3	2/3	5,7,/	7,6,/	2/3 (640, 320, <10)
HK1/68	3/3	3/3	1,1,2	6,6,6	3/3 (1,280, 1,280, 1,280)	3/3	3/3	1,2,2	7,6,6	3/3 (1,280, 1,280, 1,280)

a dpc, day postcontact.

**FIG 4:**
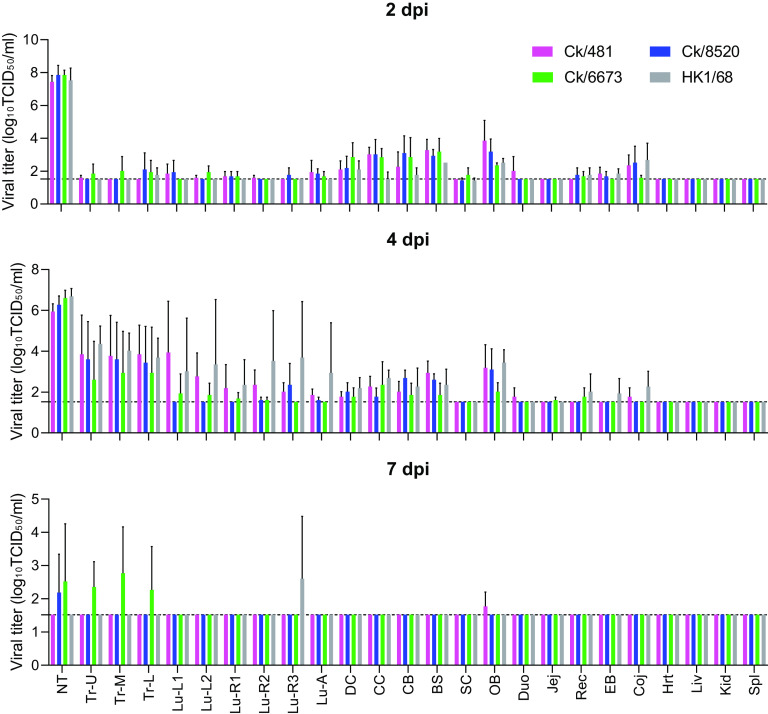
Viral distribution of H3N8 and H3N2 virus in ferrets. Ferrets were euthanized at 2, 4 and 7 dpi. The viral titers of each tissue are expressed as log_10_ TCID_50_/mL. Arithmetic mean of transformed titers with standard derivation (SD) is shown. The dashed lines indicate the detection limit at 1.52 log_10_TCID_50_/mL. (Tissues: NT, nasal turbinate; Tr, trachea; Lu, lung; DC, diencephalon; CC, cerebral cortex; CB, cerebellum; BS, brain stem; OB, olfactory bulb; SC, spinal cord; Duo, duodenum; Jej, jejunum; Rec, rectum; EB, eyeball; Coj, conjunctiva; Spl, spleen; Kid, kidney; Liv, liver; Hrt, heart).

To evaluate viral pathogenicity, ferrets (*n* = 36, nine per virus group) inoculated with the H3N8 or HK1/68 viruses were euthanized on 2, 4, and 7 dpi. Ferrets inoculated with one of the H3N8 viruses exhibited similar pathogenicity to those inoculated with HK1/68. All H3 viruses were detected primarily in the respiratory tract, with the highest loads in the nasal turbinate (Fig. 4) and lower loads in the trachea and lung tissues at 2 dpi. With the progression of virus replication, higher titers of H3N8 viruses (ranging from 10^2^ to 10^4^ TCID_50_/mL) were detected in the lower respiratory tracts (trachea and lungs) while the virus titers of nasal turbinate decreased at 4 dpi, indicating virus dissemination from upper to lower respiratory tracts. At 7 dpi, only low titers of H3 viruses were detected in respiratory tracts (Fig. 4). Viable H3 viruses were detectable in the brain and olfactory bulb at 2 and 4 dpi with titers from 10^2^ to 10^4^ TCID_50_/mL but not in the kidney, liver, heart, or spleen (Fig. 4).

**FIG 5:**
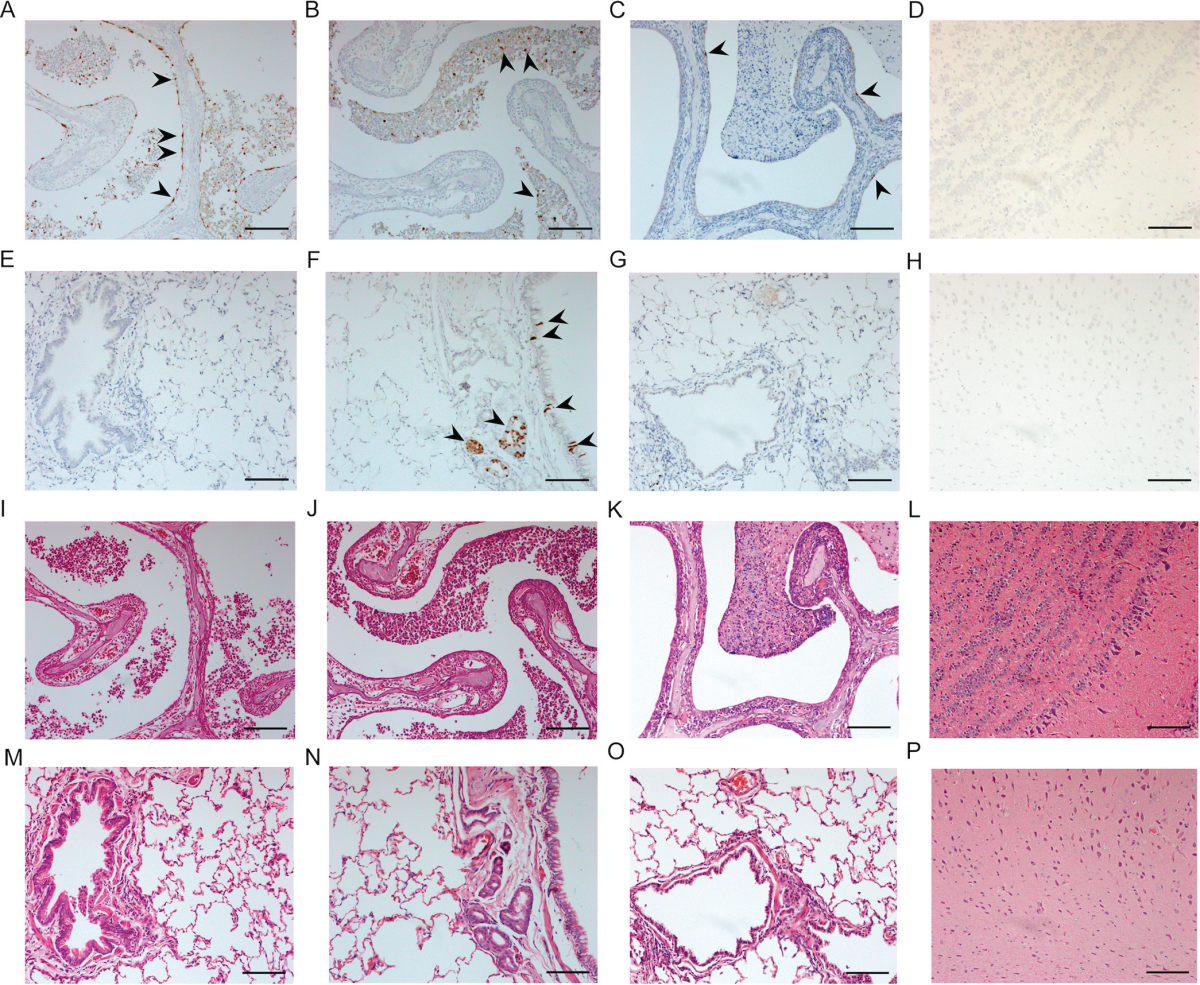
Histopathological examination of respiratory tracts and brains of ferrets infected with avian H3N8 virus. The tissues were collected at 2, 4, and 7 dpi. The pathological changes and NP expressions were similar among three H3N8 viruses. The NP expression and pathological change in above tissues of ferrets infected with Ck/8520 are shown. (A to C) NP-positive cells in nasal turbinate at 2 dpi (A), 4 dpi (B), and 7 dpi (C). (D) The NP antigen was absent in olfactory bulb at any time point (data of 4 dpi was shown). (E to G) The NP-positive cells were detected in submucosal glands and epithelial cells in the bronchus of ferrets at 4 dpi (F) but could not be detected at 2 dpi (E) and 7 dpi (G). (H) The NP antigen was absent in brains at any time point (data of cerebral cortex at 4 dpi was shown). (I to K) H&E staining of nasal turbinate at 2 dpi (I), 4 dpi (J), and 7 dpi (K). (L) No pathological change was observed in olfactory bulb at any time point (H&E staining at 4 dpi was shown). (M to O) H&E staining of lungs at 2 dpi (M), 4 dpi (N), and 7 dpi (O). (P) No pathological change was observed in brain at any time points (H&E staining of cerebral cortex at 4 dpi was shown). The pictures of the same time point were collected from the same animal. NP-positive cells are indicated by black arrows. All scale bars = 100 μm.

Hematoxylin and eosin (H&E) staining and immunostaining of influenza nucleoprotein (NP) antigen were performed in the respiratory tract and brain tissues to investigate the viral antigen distribution and pathological changes. NP was widely observed in the epithelial cells of the nasal mucosa and exudate in nasal cavities at 2 and 4 dpi (Fig. 5A and B) and largely decreased at 7 dpi (Fig. 5C). H&E staining of nasal turbinate showed that antigen-positive cells started to detach from the epithelial layer at 2 dpi (Fig. 5I), and large amounts of exudate of inflammatory cells, mucus, and exfoliated epithelial cells were seen in the nasal cavity at 4 dpi (Fig. 5J) and 7 dpi (Fig. 5K), which caused robust nasal discharge of infected ferrets. Limited amounts of NP were detected in the epithelial cells of the bronchus and submucosal glands at 4 dpi (Fig. 5F) but not at 2 dpi (Fig. 5E) and 7 dpi (Fig. 5G). No significant pathological changes were present in the bronchioles and alveoli of ferrets infected with H3N8 viruses at any time points (Fig. 5M to O). Although H3N8 or H3N2 viruses were present in brain and olfactory bulb tissues at low virus titers, there were no visible NP or pathological changes (Fig. 5D, H, L, and P).

### Adaptative substitutions emerged during infection and transmission of chicken H3N8 viruses in ferrets.

To identify potential mammalian-adaptive mutations acquired during replication and transmission of chicken H3N8 virus in ferrets, virus inoculum and a total of 28 nasal washes (selected from those with the highest load) were sequenced. Several substitutions located in different viral proteins were detected (Fig. S5). The most dominant substitution was G228S in HA, which was found in 17 of 28 specimens with partial or complete acquisition. The next dominant substitution was NS2-F116V, which was identified in all nasal washes but with only 3 to 13% enrichment compared to the inoculum. Other substitutions in the RNP complex and NS2 also emerged, but these were variably detected in the three H3N8 viruses. For Ck/481, PB2 V292G and V295G changes were found in 8/8 samples with ~10% enrichment and the E627K mutation was found with higher frequency (9 to 66.8%) in 6/8 specimens. The S17L mutation in NS2 was also found in 6/8 specimens at similar frequencies (6.5 to 53.55%). Other substitutions in PB2, PB1, and NP were found with different ranges of frequencies (Fig. S5A). For Ck/8520, fewer substitutions were identified compared to Ck/481. The HA-G228S substitution was the most common with less frequent changes observed in PB2, PB1, and HA (Fig. S5B). For Ck/6673, the most abundant substitutions were Q226L and G228S in the hemagglutinin (Fig. S5C). Notably, the HA-228S and PB2-627K residues identified in some viruses upon growth and limited transmission in ferrets were consistent with one of the human isolates (Henan/4-10/2022), suggesting the important role of these two substitutions for the avian H3N8 virus to adapt in mammalian host species.

### Prevalence of anti-H3 influenza virus antibodies in healthy humans.

Human sera, obtained from 498 healthy volunteers aged 15 to 83 years, were examined for specific antibodies against the chicken H3N8 viruses by hemagglutination inhibition (HI) and microneutralization (MN) assays. All the sera were divided into three age groups, <18, 18 to 49, and ≥50. Across all age groups, 193 and 220 sera were seropositive (HI titer ≥40) for HK/1/68 and A/Darwin/6/21, a seasonal H3N2 vaccine strain recommended by World Health Organization (WHO), respectively (Table 4). For the three selected H3N8 viruses, 23, 28, and 14 sera were seropositive (HI titer ≥40) for Ck/481, Ck/8520, and Ck/6673, respectively (Table 4). Furthermore, in the 18 to 49 age group, only 0.6% to 1.1% of sera exhibited seropositivity to the three H3N8 viruses. In contrast, 8.1% to 12.8% seropositivity was detected in the ≥50 age group, indicating people born before 1968 or shortly after have more cross-reactive antibodies to the avian H3N8 viruses.

**TABLE 4:** Prevalence of anti-H3 influenza virus antibodies in healthy humans

Assay	Age group	Total no. of sera	Titer	No. of sera (%)				
				HK1/68	Dar6/21^*d*^	Ck/481	Ck/8520	Ck/6673
HI (*n* = 498)^*a*^	<18	1	Neg.^*c*^	1	0	1	1	1
			≥40	0	1	0	0	0
	18−49	349	Neg.	286	201	345	343	347
			≥40	63 (18%)	148 (42%)	4 (1%)	6 (2%)	2 (1%)
	≥50	148	Neg.	18	77	129	126	136
			≥40	130 (87%)	71 (48%)	19 (13%)	22 (15%)	12 (8%)
	All age group	498	Neg.	305	278	475	470	484
			≥40	193 (39%)	220 (44%)	23 (5%)	28 (6%)	14 (3%)
MN (*n* = 129)^*b*^	18−49	54	Neg.	42	53	54	54	54
			≥40	12 (22%)	1 (2%)	0	0	0
	≥50	75	Neg.	5	73	73	70	73
			≥40	70 (93%)	2 (3%)	2 (3%)	5 (7%)	2 (3%)
	All age group	129	Neg.	47	126	127	124	127
			≥40	82 (64%)	3 (2%)	2 (2%)	5 (4%)	2 (2%)

a HI, hemagglutination inhibition assay.

b Sera had a hemagglutination inhibition (HI) titer ≥10 against H3N8 viruses subjected to microneutralization (MN) test (*n* = 129).

c Negative, HI, or MN titers <40.

d Dar6/21 or Darwin/6/2021.

Sera that exhibited an HI titer ≥10 (*n* = 129) to any of the chicken H3N8 viruses were tested in an MN assay, and only 2, 5, and 2 sera from the ≥50 age group showed titers ≥40 to Ck/481, Ck/8520, and Ck/6673, respectively (Table 4). Five sera that were positive against the H3N8 virus, with neutralizing titers ranging from 10 to 640, had titers at least 4-fold higher for HK1/68 (Table 5).

**TABLE 5:** Neutralizing titer of five seropositive human sera

Serum no.^*a*^	Age	MN titer				
		HK1/68	Dar6/21	Ck/481	Ck/8520	Ck/6673
1	52	160	<10	10	40	10
2	53	320	<10	20	40	10
3	59	320	<10	20	40	10
4	55	5,120	80	80	160	40
5	57	2,560	<10	160	640	160

a Five human sera that were seropositive against any chicken H3N8 viruses.

## DISCUSSION

This study aimed to investigate the genesis and emergence of a recently identified zoonotic H3N8 virus. Based on the phylogenetically closest viruses, our surveillance data showed that the H3N8 virus most likely originated in the Guangxi-Guangdong region of China. We were also able to show that the H3N8 viruses had been circulating in chickens for over a year at a high prevalence and had disseminated to at least seven provinces before detection in humans (Fig. 1). We detected an obvious seasonality that was also observed in the H7N9/2013 influenza viruses (10). Like the H7N9/2013 and H10N8/2013 viruses, the H3N8 viruses mixed their duck-origin HA and NA genes with internal genes from chicken H9N2 viruses enzootic in the region (5, 8, 10, 11). This demonstrates again that the influenza ecosystem of China, with its high and frequently interacting human and poultry populations, is a major source of novel influenza viruses with zoonotic capacity.

Phylogenetic evaluation of the sequences we generated showed that the chicken H3N8 viruses continue to evolve and undergo frequent reassortment with contemporary enzootic H9N2 viruses (Fig. 2 and Fig. S1 and S2), which might lead to optimization of its fitness in chickens. Given its current low pathogenicity toward chickens (24), control measures against this virus in poultry are unlikely to be initiated based on agricultural considerations. Without interventions, subsequent epidemic waves will likely occur in the coming winters, as seen with H7N9 viruses until vaccination of poultry was introduced (10, 25). While there are unlikely to be agricultural drivers of control measures, as human infections with the H3N8 virus have already occurred, public health pressures may influence vaccination discussions.

H3 viruses are one of only three influenza subtypes to have caused a pandemic, and the H3 subtype has shown the greatest propensity to infect mammals (18, 21, 22). Apart from sporadic infections in aquatic mammals (19) and carnivores (26), this subtype has become established in swine, equids, and canids (18, 27, 28). Of the persistent mammalian H3 lineages, three are of whole or partial avian origin, with the human and canine H3N2 lineages emerging in China (18). As H3 is a major influenza subtype in the wild birds in China, monitoring and control of H3 viruses entering the agricultural or human environments seem advisable.

Our *in vivo* infection and transmission studies demonstrated that the chicken H3N8 virus can infect ferrets and transmit, albeit inefficiently by the airborne route, among ferrets. Adaptative mutations (HA G228S and PB2 E627K) associated with increased infectivity and transmissibility of avian virus in mammals had emerged from infected ferrets and even in some recipient ferrets. These adaptations were also found in one of the human H3N8 viruses, suggesting that human-to-human transmission of the H3N8 virus might occur. Current antibody levels in the human population, as inferred from our serology survey, will not provide sufficient immune protection against infection with this virus. Thus, if this H3N8 virus continues to persist and expand in poultry, opportunities for its spillover to humans may be greatly increased. The similarly derived H7N9 virus caused more human infections in the more extensive second wave of its outbreak (10).

Whether this chicken H3N8 influenza could develop into a pandemic pathogen is an important question. Further transmissions to humans, as well as reassortment with circulating seasonal influenza viruses, are scenarios that could result in serious consequences, as similar events occurred to form the H3N2/68 and H2N2/57 pandemic viruses (29, 30). Although the first H3N8 human infection case developed severe disease, the second human case and the ferrets infected with the chicken H3N8 viruses exhibited slight upper respiratory symptoms and recovered rapidly from the infection. This suggests that this H3N8 virus may have a low capacity to cause disease in uncomplicated cases in humans (13–15). Zoonotic infections that might occur could, therefore, be misdiagnosed as seasonal influenza, which could make monitoring any human adaptations difficult unless sufficient surveillance is put in place.

The emergence of this novel H3N8 virus highlights again the persistent threat posed by the increasingly complicated landscape of influenza viruses in China. Its acquisition of internal genes from enzootic H9N2 viruses was seen in the H7N9 and H10N8 zoonotic viruses and is evidence that H9N2 viruses in poultry may play a “facilitator” role in bringing wild bird influenza subtypes to poultry (5, 8, 10, 11, 31, 32). If the avian origin segments obtained to create the 1957 and 1968 pandemic viruses were first transferred to poultry, then the current influenza ecosystem in China with enzootic H9N2 viruses may be a comparatively more favorable field to transfer avian influenza virus segments to humans. Whether the current H3N8 viruses will continue to circulate in poultry or cause further human infections is likely to be determined by the type, strength, and timing of control measures applied in the field. As this virus already has a wide geographic distribution but currently shows low pathogenicity in chickens and mammals, passive and active surveillance of both animal and human populations should be undertaken as a matter of urgency.

## MATERIALS AND METHODS

### Influenza surveillance in southern China.

Findings from influenza surveillance of chickens and silkie chickens from LPMs in six provinces of southern China (Jiangxi, Guangdong, Guangxi, Fujian, Guizhou, and Yunnan) from January 2021 to September 2022 are summarized in Table 1 and Fig. 1. Oropharyngeal and cloacal swabs were taken from market birds and immediately preserved in viral transport medium with antibiotics and held in 4°C cool boxes before being sent to the laboratory. All samples were inoculated into 9- to 10-day-old embryonated chicken eggs and incubated for 48 to 72 h at 37°C to isolate influenza viruses. After dipping, hemagglutinin-positive allantoic fluids were harvested and subtyped by HI assays using a panel of WHO reference antisera. All operations followed WHO guidelines on Animal Influenza Diagnosis and Surveillance (33).

### Whole-genome sequencing.

A total of 534 H3 HI-positive isolates were sequenced as described below. Viral RNA was extracted from these samples using QIAamp Viral RNA minikit (Qiagen). cDNA was synthesized using PrimeScript II 1st Strand cDNA Synthesis kit (TaKaRa Bio) with influenza-specific primers as previously described (34). A DNA library was constructed using the TruePrep DNA Library Prep kit V2 for Illumina (Vazyme) and sequenced by a Mi-Seq desktop sequencer (Illumina) using MiSeq Reagent Kits v3 (Illumina) according to the manufacturer’s instructions.

PRINSEQ was used to remove low-quality and short raw reads. Then, 3,000 of these preprocessed reads were randomly selected and assembled into contigs with overlapping regions of 40 nucleotides and >90% identity using the GS De Novo Assembler (version 2.6, Roche). BLASTn was used to search the contigs against the influenza A virus sequences available at GenBank, and the most similar sequence was used as the template to assemble the rest of the preprocessed reads via reference assembly using MIRA. Samples containing more than one copy of a gene with <97% sequence identity were considered mixed infections and were removed before phylogenetic analysis. Whole genomes of 274 unmixed H3N8 chicken viruses obtained in this study were submitted to GenBank. The accession numbers are OQ291618 to OQ293841.

### Phylogenetic analysis.

All complete genomes of influenza A viruses were downloaded from GISAID (http://www.gisaid.org) on 13 May 2022. Sequences with ambiguous bases (>0.5%) or too short length (<70% full length) were removed from the data sets. The retained sequences were combined with the sequences generated in this study, and multiple sequence alignments were performed for each gene segment using MAFFT v7.273 (35). The resulting alignments were manually adjusted for subsequent phylogenetic analysis.

Global phylogenetic trees for each gene segment were first estimated using the fast maximum likelihood method in FastTree v2.1.11 (36) to identify the major lineage containing the two zoonotic H3N8 viruses (A/Henan/4-10/2022 and A/Changsha/1000/2022) recently reported in China. Viruses closely related to the lineage containing the zoonotic H3N8 viruses were extracted and combined with reference sequences according to the subtype, host, and geographic location. Maximum likelihood phylogenies were constructed from these refined data sets using the GTR-Γ_4_-I model and 1,000 bootstrap replicates using IQ-TREE v1.6.12 (37). The HA, NA, and PB2 gene trees of selected viruses are presented in Fig. 2 and full trees for all segments are presented in Fig. S1 and S2. Acknowledgment of the contributors of the GISAID sequences used in these phylogenetic trees is given in Data set S1.

### Cells and viruses.

Madin-Darby canine kidney (MDCK) cells were obtained from the American Type Culture Collection (ATCC; CCL-34) and maintained in minimum essential media (MEM, Gibco) with 10% fetal bovine serum (FBS; Gibco). Three viruses, Ck/Guangdong-Shantou/481/2022 (Ck/481), Ck/Jiangxi/8520/2022 (Ck/8520), and Ck/Guangdong-Shantou/6673/2021 (Ck/6673), were selected, one per subclade, from the H3N8 zoonotic lineage. Stocks of these viruses were prepared and purified by three continuous passages in MDCK cells by plaque assays. Two reference human H3N2 influenza viruses, A/Hong Kong/1/1968 (HK1/68) and A/Darwin/6/2021 (Darwin6/21), were obtained from WHO and Dayan Wang of the Chinese Centre for Disease Control and Prevention, respectively. After being passaged twice, their stocks were also prepared in MDCK cells. All supernatants were harvested and stored in aliquots at −80°C until use. The virus stocks were titrated by standard TCID_50_ assays, and their full genomes were confirmed by next-generation sequencing.

### Animals and ethical statement.

Eight- to nine-month-old ferrets (both male and female, Mustela putorius furo) were purchased from Wuxi Sangosho Co. Ltd. and were confirmed to be influenza-free by HI assays against human seasonal influenza (H1N1, H3N2, and B/Victoria-like) viruses and contemporary avian H9N2 and H3N8 viruses related to this study. Nasal washes were taken for virus isolation in MDCK cells to confirm that no influenza infection occurred during transportation. All animal experimental protocols were approved by the Institutional Ethical Review Board of Shantou University Medical College (SUMC2022-258). All the animal infections were conducted in biosafety level 3 (BSL3) containment facilities using practices in accordance with the approved institutional guidelines as previously described (38).

### Replication and pathogenicity in ferrets.

Nine male ferrets per virus group were intramuscularly anesthetized by tiletamine-zolazepam-xylazine (1 mg/kg body weight) and inoculated intranasally with 10^6^ TCID_50_ virus in 0.5 mL of MEM (39). Three ferrets from each virus group were humanely euthanized at 2, 4, and 7 dpi to collect all major organs: the nasal turbinate, trachea (the upper, middle, and lower parts were collected separately), lung (each lobe was collected separately), brain (the cerebral cortex, diencephalon, cerebellum, and brain stem were collected separately), olfactory bulb, spinal cord, intestine (the duodenum, jejunum, and rectum were collected separately), eyeball, conjunctiva, spleen, kidney, liver, and heart. To determine the virus load, 0.5 × 0.5-cm pieces of tissue were collected in 1 mL cold phosphate-buffered saline (PBS) with 4% penicillin (100 U/mL) and streptomycin (100 μg/mL) and subsequently stored at −80°C until further analysis. During necropsy, the surgical instruments were disinfected with 75% ethanol to prevent cross-contamination among different tissues. Frozen tissues were thawed and homogenized by Qiagen TissueLyser II. Solid debris was pelleted by centrifugation and the supernatant was titrated by a standard TCID_50_ assay.

For pathological examinations, tissue cubes (1 × 1 × 1 cm) were fixed in 10% neutral buffered formalin (NBF) (after inflation with formalin for the lung) for 3 days. The fixed cubes were removed from formalin and then processed for routine paraffin embedding (40). Three-micrometer serial sections were mounted on poly-l-lysine glass slides for H&E and immunohistochemistry (IHC) staining. For the nasal cavity, the lower jaw and skin or muscle around the nose were removed. One side of the nose tissues was fixed in 10% NBF and subjected to decalcification using PBS with 10% formic acid and 10% hydrochloric acid. After decalcification, tissues were processed for paraffin embedding.

### IHC staining.

For detection of NP antigen, the 3-μm deparaffinized slides were subjected to heat-induced epitope retrieval in a pressure cooker for 2 min and incubated with nonspecific binding blocking solution (PBS with 3% bovine serum albumin) for 30 minutes. The slides were further stained with primary monoclonal antibody against influenza NP antigen, which was kindly provided by Yixin Chen from Xiamen University (41). The binding of primary antibodies was recognized by a secondary anti-mouse antibody labeled with biotin (BOSTER) and further labeled with horseradish peroxidase (HRP; MXB Biotechnologies). 3,3′-Diaminobenzidine (DAB; MXB Biotechnologies) was used as the substrate to visualize NP-positive cells in tissues.

### Transmission in ferrets.

For the direct-contact transmission study, four groups of female ferrets (*n* = 3) were anesthetized as above and intranasally inoculated with 10^6^ TCID_50_ viruses in 0.5 mL of MEM (Fig. S4A). Three naive female ferrets for each group were transferred to be cohoused with inoculated ferrets on 1 dpi. For the airborne transmission study, experiments were performed within bioisolators with directional airflow control (Fig. S4B). The airflow rates were adjusted to >0.1 to approximately <0.3 m/s and the temperature and humidity were set at 22°C and 60 to 70%. Four groups of male ferrets (*n* = 3) were anesthetized and intranasally inoculated with 10^6^ TCID_50_ viruses in 0.5 mL of MEM to serve as donors for the airborne transmission experiments. A naive male ferret was housed in a separate cage, 10 cm apart, on 1 dpi for each donor ferret. The airborne-exposed naive animals were operated on first, with an independent set of tools, to avoid contamination from inoculated animals.

All the animals in direct-contact and airborne transmission groups were monitored for clinical signs and nasal washes were collected daily, for 14 days, to detect the presence of infectious virus, as previously described (38). Clinical symptoms, such as sneezing, nasal discharge, weight change, temperature change, and level of inactivity, were monitored once or twice per day. Temperature was measured twice daily using a subcutaneous implantable temperature transponder (IPTT-300, Bio Medic Data Systems). A scoring standard was used to evaluate the activity level: 0, alert and playful; 1, alert but playful only when stimulated; 2, alert but not playful when stimulated; and 3, neither alert nor playful when stimulated (42). The relative inactivity score was calculated as previously described (43). Temperature, body weight, and activity were observed for 3 days before infection to obtain baseline values. Three animals, used as controls, were inoculated with MEM without a virus in an equivalent volume to that used for the virus-inoculated ferrets. Seroconversion was determined by an HI assay using sera collected at 14 dpi (or dpc) and 21 dpi (or dpc).

### Next-generation sequencing and genetic analyses of inoculums and ferret specimens.

Inoculums and nasal wash specimens collected from ferrets in transmission studies were all subjected to whole-genome sequencing, using the same procedure mentioned above. Reads generated by Illumina sequencing were preprocessed by PRINSEQ. These reads were then assembled by MIRA using the coding regions of the inoculums as references. Amino acid variation on each position of the influenza proteins was calculated according to these assembled reads.

### Sera collection and ethical approval.

Human sera were collected in August 2022 from 498 healthy volunteers, aged 15 to 83, in the Physical Examination Center of the First Affiliated Hospital of Shantou University Medical College (Guangdong, China). The sera were used to detect the presence of antibodies against H3 influenza viruses. All procedures were approved by the ethical committee of the First Affiliated Hospital of Shantou University Medical College, and all volunteers were informed about the procedures and risks before enrollment in the study. The serum samples were collected and stored at −20°C in aliquots until further analysis.

### Serological studies.

HI and microneutralization (MN) assays were conducted as previously described (44). Human serum samples were treated with receptor destroying enzyme II (RDE II; Denka Seiken) to remove nonspecific inhibitors, and further nonspecific agglutinins were removed by treating with turkey red blood cells (RBCs). The sera were diluted to 1:10 and screened against three chicken H3N8 strains (Ck/481, Ck/8520, and Ck/6673), the current human seasonal H3N2 virus (Darwin6/21), and the human pandemic H3N2 virus (HK1/68) by HI assays to detect the presence of anti-H3 influenza virus antibodies. Serum samples that reacted to H3N8 viruses with an HI titer ≥10 were used to detect neutralizing antibodies against the H3N8 viruses Darwin6/21 and HK1/68 by MN assays. Full results are given in Data set S2.

### Data availability.

Genome sequences obtained in this study were submitted to GenBank, under the accession numbers OQ291618 to OQ293841.
