# Supplementary material for: Characterization of an Emergent Chicken H3N8 Influenza Virus in Southern China: a Potential Threat to Public Health
Source: J Virol. 2023 Jun 8;97(6):e00434-23. doi: 10.1128/jvi.00434-23 (PMC10308888; doi:10.1128/jvi.00434-23)
Supplement: Supplemental file 3 — Fig. S1 to S5 and Table S1. Download jvi.00434-23-s0001.docx, DOCX file, 2.9 MB [file jvi.00434-23-s0001.docx]

­­­­­­­ **­**­­­­­­ ­­

**FIG. S1.** Maximum-likelihood phylogenies of the surface protein genes. (A) HA gene (H3, n=481); (B) NA gene (N8, n=414). The trees were constructed using IQTREE. The recent human H3N8 viruses are labeled in red and the viruses isolated in this study are colored in blue (abbreviated names). The novel chicken H3N8 lineage is highlighted with a green background. Equine/Uruguay/1/1963-like equine H3N8 lineage was used as an outgroup to root the tree. Bootstrap values from 1,000 pseudo-replicates are shown on selected branches. The length of the scale bar corresponds to 0.1 nucleotide substitution per site. Abbreviations for host species: Ck (Chicken), SCk (Silkie chicken), Dk (Duck), Qa (Quail), Pa (Partridge), Gs (Goose), W.f. Gs (White-fronted goose), N. pintail (Northern pintail), MDk (Muscovy duck). Geographical abbreviations: JX (Jiangxi), GD (Guangdong), ST (Shantou), DG (Dongguan), GZ (Guangzhou), HZ (Huizhou). Readers can zoom in to view the strain names with clarity.

­­

**FIG. S2.** Maximum-likelihood phylogeny of the internal genes. (A) PB2 tree, n=703; (B) PB1 tree, n=707; (C) PA tree, n=717; (D) NP gene, n=716; (E) MP gene, n=719; (F) NS gene, n=718. The trees were constructed using IQTREE. The recent human H3N8 viruses are labeled in red and the viruses isolated in this study are colored in blue (abbreviated names). The enzootic H9N2 lineages are highlighted in orange boxes. Avian gene pool viruses from Eurasia or North American, are indicated. The Equine/Uruguay/1/1963-like equine H3N8 lineage was used as an outgroup to root the tree, except for the NS tree will is mid-point rooted to separate alleles A and B. Bootstrap values from 1,000 pseudo-replicates are shown on selected branches. The length of the scale bar corresponds to 0.1 nucleotide substitution per site. Abbreviations for host species: Ck (Chicken), SCk (Silkie chicken), Dk (Duck), Qa (Quail), Pa (Partridge), Gs (Goose), W.f. Gs (White-fronted goose), N. pintail (Northern pintail), MDk (Muscovy duck). Geographical abbreviations: JX (Jiangxi), GD (Guangdong), ST (Shantou), DG (Dongguan), GZ (Guangzhou), HZ (Huizhou). Readers can zoom in to view the strain names with clarity. Allele A and B are indicated with brackets in NS gene tree.

**
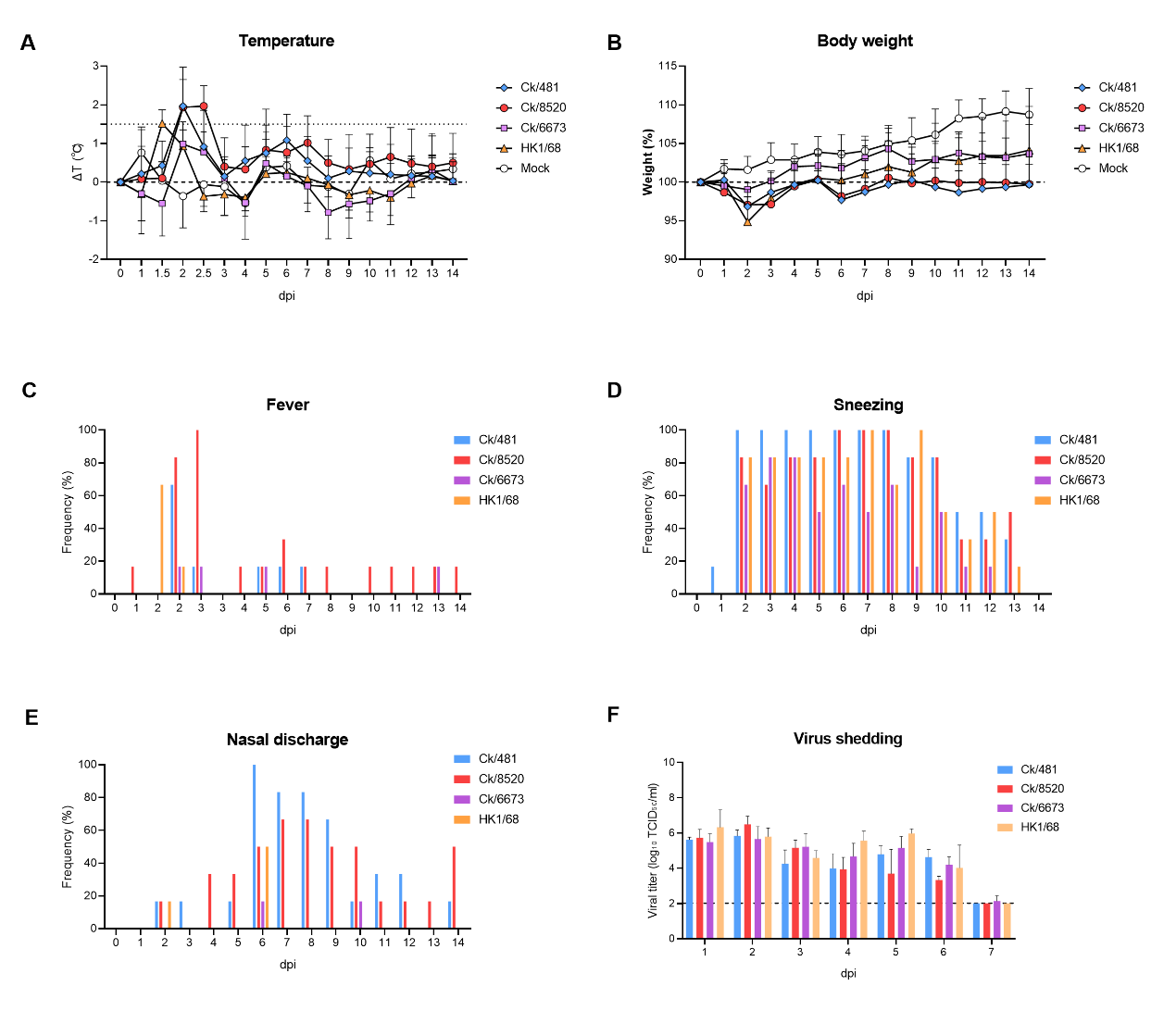
**

**FIG. S3.** Clinical symptoms observed in ferrets inoculated with H3N8 and H3N2 viruses. **(A)** Temperature change relative to that prior to infection is shown as Delta Temperature **(**ΔT). **(B)** Body weight is expressed as percentage of weight at 0 dpi with mean and standard deviation shown. **(C)** Percentage of ferrets representing with fever, defined as ≥1.5℃ higher than baseline. **(D)** Percentage of ferrets having a nasal discharge. **(E)** The percentage of ferrets sneezing. **(F)** Virus shedding, measured as log_10_ TCID_50_/ml from nasal washes collected from each ferret on 1-7 dpi. The dashed line is the detection limit, 2.02 log_10_ TCID_50_/ml.

**
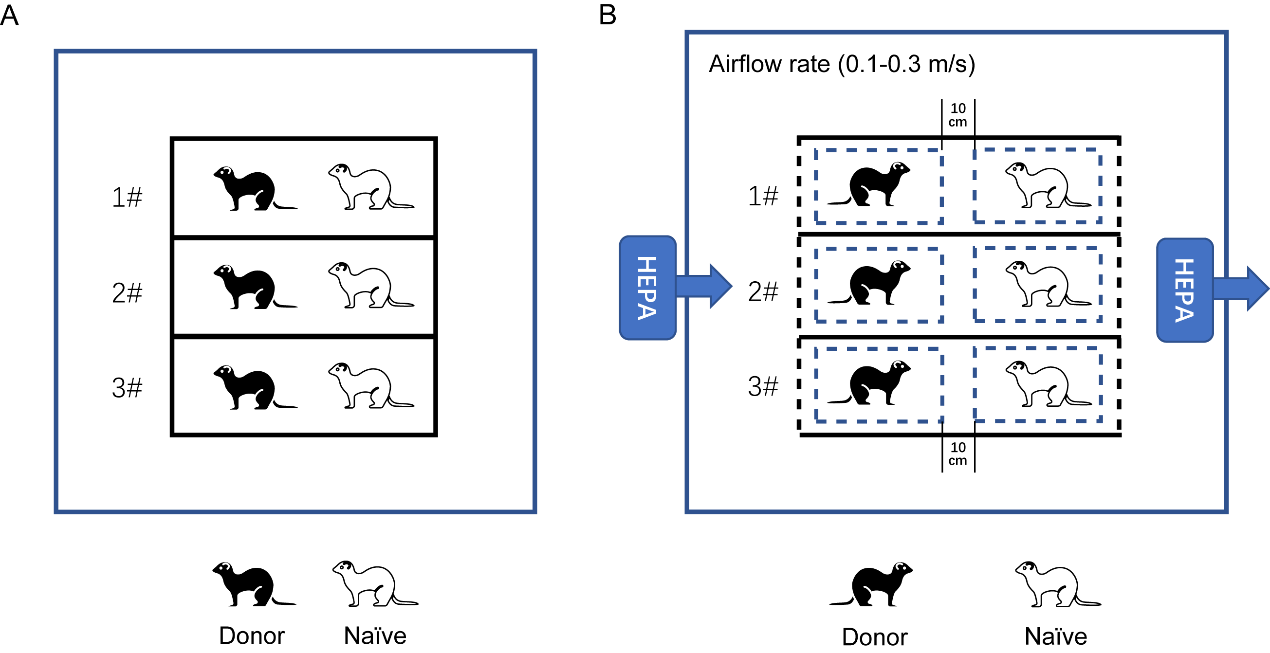
**

**FIG. S4.** Schematic diagram of ferret transmission studies. A vertical view of the transmission design is shown. Donor ferrets (black) were intranasally inoculated with 10^6^ TCID_50_/ml viruses of Ck/481, Ck/8520, Ck/6673 and HK1/68. **(A)** Each direct-contact ferret was housed in the same cage as an inoculated ferret on 1dpi. **(B)** Airborne-exposed ferrets were introduced into cages, separated by 10 cm from a caged donor ferret, on 1 dpi. The airflow rate was adjusted to >0.1 ~ <0.3 m/s. There were three one-to-one transmission pairs for each virus and each transmission type.


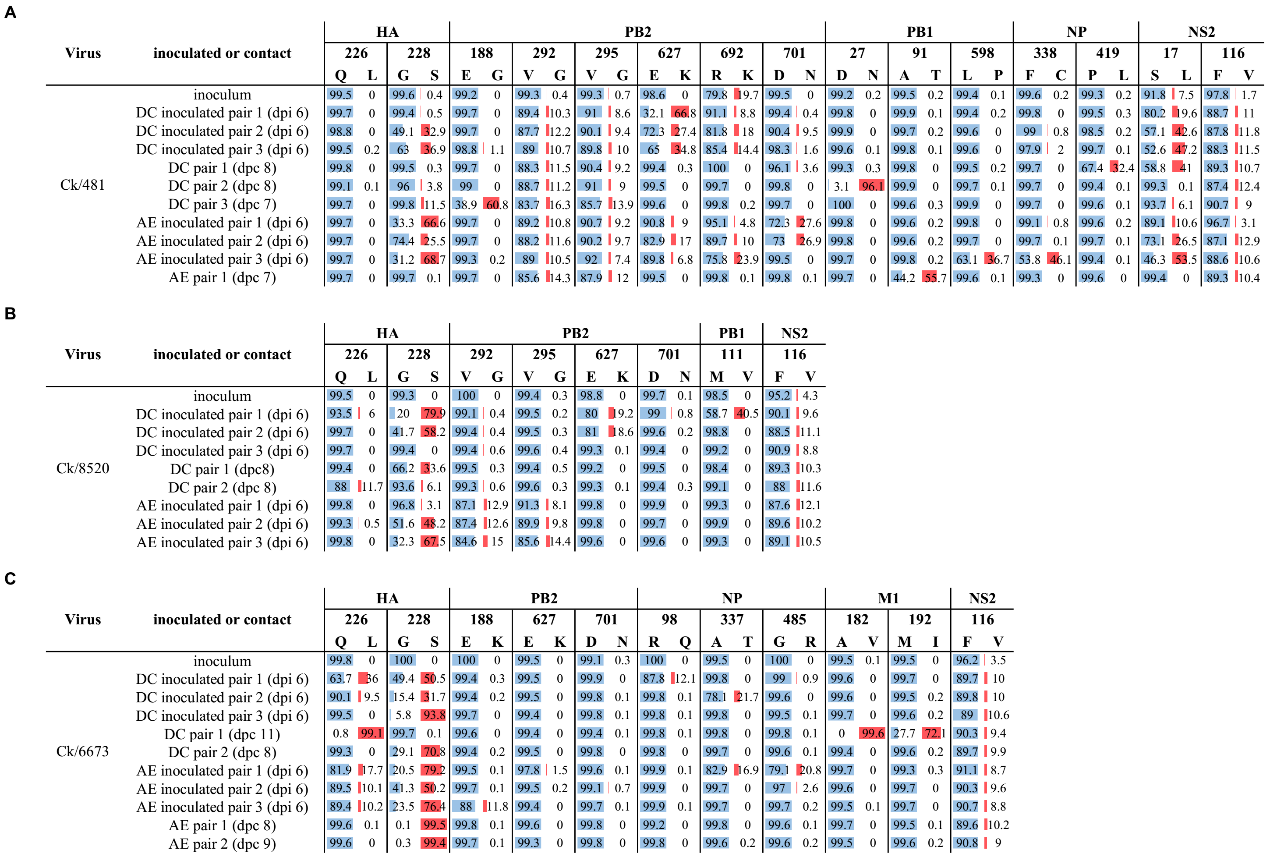


**FIG. S5**. Percentage of minor variants observed in inoculums and ferret specimens. The nasal washes collected at 6 dpi from inoculated group ferrets and peak titer nasal washes of direct-contact (DC) group or airborne-exposed (AE) ferrets were subjected for deep sequencing. Percentage of minor variants of each specimen and inoculum is shown. The percentage of original amino acid residue is displayed on the left with blue bar while the change one is on the right with red bar. Shared minor variants of three virus group at levels above the 10% cutoff threshold were present. (**A**) Percentage of minor variants of specimens from Ck/481 infected ferrets. (**B**) Percentage of minor variants of specimens from Ck/8520 infected ferrets. (**C**) Percentage of minor variants of specimens from Ck/6673 infected ferrets.

**TABLE S1**. Examination of mammalian adaptation markers of chicken H3N8 viruses.

| Protein | Mammalian adaptation marker | | Amino acid | chicken H3N8 | human H3N8 |
| --- | --- | --- | --- | --- | --- |
| HA^a^ | Increase binding to mammalian-like receptor (1–4) | G186V | G | 0 | 0 |
|  |  |  | V | 0 | 0 |
|  |  |  | S | 274 | 2 |
|  |  | Q226L | Q | 274 | 2 |
|  |  |  | L | 0 | 0 |
|  |  | G228S | G | 274 | 1 |
|  |  |  | S | 0 | 0 |
|  |  |  | G/S | 0 | 1 |
|  | Decrease HA activation pH (5–9) | HA1-H17Y | H | 274 | 2 |
|  |  |  | Y | 0 | 0 |
|  |  | HA2-K51A/E | K | 274 | 2 |
|  |  |  | A/E | 0 | 0 |
|  |  | HA2-K58I | K | 274 | 2 |
|  |  |  | I | 0 | 0 |
|  |  | HA2-T156N | T | 274 | 2 |
|  |  |  | N | 0 | 0 |
| PB2 | Increase viral polymerase function and attenuates IFN-β induction in human cells (10) | I292V | I | 28 | 0 |
|  |  |  | V | 243 | 2 |
|  | Enhance polymerase activity, virus replication and virulence in mice (11) | A588V | A | 5 | 0 |
|  |  |  | I | 7 | 0 |
|  |  |  | V | 262 | 2 |
|  | Increase virulence in mammals (12) | V598T/I | V | 274 | 2 |
|  |  |  | T/I | 0 | 0 |
|  | Enhance polymerase activity and replication in mammalian cells (13) | E627K | E | 274 | 1 |
|  |  |  | K | 0 | 1 |
|  | Enhance polymerase activity and replication in mammalian cells (14) | D701N | D | 274 | 2 |
|  |  |  | N | 0 | 0 |
|  | Species-specific signature (15) | K702R | K | 2 | 1 |
|  |  |  | R | 272 | 1 |
| PB1 | Confer higher polymerase activity in mammalian cells (16) | L473V | L | 0 | 0 |
|  |  |  | V | 274 | 2 |
|  | Correlate with transmissibility in ferrets (4) | I368V | I | 2 | 0 |
|  |  |  | V | 272 | 2 |
| PA | Increase mammalian replication and pathogenicity (17) | K356R | K | 0 | 0 |
|  |  |  | R | 274 | 2 |
|  | Species-specific signature (15) | S409N | S | 0 | 0 |
|  |  |  | N | 274 | 2 |
| NS1 | Enhance CPSF30 binding and replication in mice (18) | I106M | I | 274 | 2 |
|  |  |  | M | 0 | 0 |
|  | Increase virulence in mice (19) | N139D | N | 274 | 2 |
|  |  |  | D | 0 | 0 |
|  | Enhance virus replication in mammalian cells, while retain replication ability in avian cells (20) | E172K | E | 0 | 0 |
|  |  |  | K | 274 | 2 |
|  | Enhance viral polymerase activity and virus replication in mammalian cells (21) | N205S | N | 0 | 0 |
|  |  |  | R | 5 | 1 |
|  |  |  | S | 269 | 1 |
| M1 | Abolish transmission of H7N9 virus in ferret and in guinea pig (22) | D156E | D | 274 | 2 |
|  |  |  | E | 0 | 0 |

a, A total of 274 chicken H3N8 viruses without mix infection with other influenza virus were analyzed. The numbering of HA protein is based on H3 numbering.

REFERENCES

1. **Dortmans JCFM**, **Dekkers J**, **Ambepitiya Wickramasinghe IN**, **Verheije MH**, **Rottier PJM**, **Van Kuppeveld FJM**, **De Vries E**, **De Haan CAM**. 2013. Adaptation of novel H7N9 influenza A virus to human receptors. Sci Rep **3**:1–7.

2. **Rogers GN**, **Paulson JC**, **Daniels RS**, **Skehel JJ**, **Wilson IA**, **Wiley DC**. 1983. Single amino acid substitutions in influenza haemagglutinin change receptor binding specificity. Nature.

3. **Vines A**, **Wells K**, **Matrosovich M**, **Castrucci MR**, **Ito T**, **Kawaoka Y**. 1998. The Role of Influenza A Virus Hemagglutinin Residues 226 and 228 in Receptor Specificity and Host Range Restriction. J Virol **72**:7626–7631.

4. **Herfst S**, **Schrauwen EJA**, **Linster M**, **Chutinimitkul S**, **De Wit E**, **Munster VJ**, **Sorrell EM**, **Bestebroer TM**, **Burke DF**, **Smith DJ**, **Rimmelzwaan GF**, **Osterhaus ADME**, **Fouchier RAM**. 2012. Airborne transmission of influenza A/H5N1 virus between ferrets. Science (80- ) **336**:1534–1541.

5. **Thoennes S**, **Li ZN**, **Lee BJ**, **Langley WA**, **Skehel JJ**, **Russell RJ**, **Steinhauer DA**. 2008. Analysis of residues near the fusion peptide in the influenza hemagglutinin structure for roles in triggering membrane fusion. Virology **370**:403–414.

6. **Steinhauer DA**, **Wharton SA**, **Skehel JJ**, **Wiley DC**, **Hay AJ**. 1991. Amantadine selection of a mutant influenza virus containing an acid-stable hemagglutinin glycoprotein: Evidence for virus-specific regulation of the pH of glycoprotein transport vesicles. Proc Natl Acad Sci U S A **88**:11525–11529.

7. **Reed ML**, **Yen H-L**, **DuBois RM**, **Bridges OA**, **Salomon R**, **Webster RG**, **Russell CJ**. 2009. Amino Acid Residues in the Fusion Peptide Pocket Regulate the pH of Activation of the H5N1 Influenza Virus Hemagglutinin Protein. J Virol **83**:3568–3580.

8. **Zaraket H**, **Bridges OA**, **Russell CJ**. 2013. The pH of Activation of the Hemagglutinin Protein Regulates H5N1 Influenza Virus Replication and Pathogenesis in Mice. J Virol **87**:4826–4834.

9. **Keleta L**, **Ibricevic A**, **Bovin N V.**, **Brody SL**, **Brown EG**. 2008. Experimental Evolution of Human Influenza Virus H3 Hemagglutinin in the Mouse Lung Identifies Adaptive Regions in HA1 and HA2. J Virol **82**:11599–11608.

10. **Gao W**, **Zu Z**, **Liu J**, **Song J**, **Wang X**, **Wang C**, **Liu L**, **Tong Q**, **Wang M**, **Sun H**, **Sun Y**, **Liu J**, **Chang KC**, **Pu J**. 2019. Prevailing I292V PB2 mutation in avian influenza H9N2 virus increases viral polymerase function and attenuates IFN-β induction in human cells. J Gen Virol **100**:1273–1281.

11. **Xiao C**, **Ma W**, **Sun N**, **Huang L**, **Li Y**, **Zeng Z**, **Wen Y**, **Zhang Z**, **Li H**, **Li Q**, **Yu Y**, **Zheng Y**, **Liu S**, **Hu P**, **Zhang X**, **Ning Z**, **Qi W**, **Liao M**. 2016. PB2-588V promotes the mammalian adaptation of H10N8, H7N9 and H9N2 avian influenza viruses. Sci Rep **6**:1–13.

12. **Hu M**, **Yuan S**, **Zhang K**, **Singh K**, **Ma Q**, **Zhou J**, **Chu H**, **Zheng BJ**. 2017. PB2 substitutions V598T/I increase the virulence of H7N9 influenza A virus in mammals. Virology **501**:92–101.

13. **Zhang H**, **Li X**, **Guo J**, **Li L**, **Chang C**, **Li Y**, **Bian C**, **Xu K**, **Chen H**, **Sun B**. 2014. The PB2 E627K mutation contributes to the high polymerase activity and enhanced replication of H7N9 influenza virus. J Gen Virol **95**:779–786.

14. **Zhu W**, **Li L**, **Yan Z**, **Gan T**, **Li L**, **Chen R**, **Chen R**, **Zheng Z**, **Hong W**, **Wang J**, **Smith DK**, **Guan Y**, **Zhu H**, **Shu Y**. 2015. Dual E627K and D701N mutations in the PB2 protein of A(H7N9) influenza virus increased its virulence in mammalian models. Sci Rep **5**:1–11.

15. **Finkelstein DB**, **Mukatira S**, **Mehta PK**, **Obenauer JC**, **Su X**, **Webster RG**, **Naeve CW**. 2007. Persistent Host Markers in Pandemic and H5N1 Influenza Viruses. J Virol **81**:10292–10299.

16. **Xu C**, **Hu W Bin**, **Xu K**, **He YX**, **Wang TY**, **Chen Z**, **Li TX**, **Liu JH**, **Buchy P**, **Sun B**. 2012. Amino acids 473V and 598P of PB1 from an avian-origin influenza A virus contribute to polymerase activity, especially in mammalian cells. J Gen Virol **93**:531–540.

17. **Xu G**, **Zhang X**, **Gao W**, **Wang C**, **Wang J**, **Sun H**, **Sun Y**, **Guo L**, **Zhang R**, **Chang K-C**, **Liu J**, **Pu J**. 2016. Prevailing PA Mutation K356R in Avian Influenza H9N2 Virus Increases Mammalian Replication and Pathogenicity. J Virol **90**:8105–8114.

18. **Ayllon J**, **Domingues P**, **Rajsbaum R**, **Miorin L**, **Schmolke M**, **Hale BG**, **García-Sastre A**. 2014. A Single Amino Acid Substitution in the Novel H7N9 Influenza A Virus NS1 Protein Increases CPSF30 Binding and Virulence. J Virol **88**:12146–12151.

19. **Huang K**, **Mao H**, **Ren P**, **Zhang Y**, **Sun X**, **Zou Z**, **Jin M**. 2022. 139D in NS1 Contributes to the Virulence of H5N6 Influenza Virus in Mice. Front Vet Sci **8**:1–9.

20. **Huang X**, **Zheng M**, **Wang P**, **Mok BWY**, **Liu S**, **Lau SY**, **Chen P**, **Liu YC**, **Liu H**, **Chen Y**, **Song W**, **Yuen KY**, **Chen H**. 2017. An NS-segment exonic splicing enhancer regulates influenza A virus replication in mammalian cells. Nat Commun **8**:1–15.

21. **Patil A**, **Anhlan D**, **Ferrando V**, **Mecate-Zambrano A**, **Mellmann A**, **Wixler V**, **Boergeling Y**, **Ludwig S**. 2021. Phosphorylation of Influenza A Virus NS1 at Serine 205 Mediates Its Viral Polymerase-Enhancing Function. J Virol **95**.

22. **Kong H**, **Ma S**, **Wang J**, **Gu C**, **Wang Z**, **Shi J**, **Deng G**, **Guan Y**, **Chen H**. 2019. Identification of Key Amino Acids in the PB2 and M1 Proteins of H7N9 Influenza Virus That Affect Its Transmission in Guinea Pigs. J Virol **94**.
